# Comorbid anxiety predicts lower odds of depression improvement during smartphone-delivered psychotherapy

**Published:** 2025-01-22

**Authors:** Morgan B. Talbot, Jessica M. Lipschitz, Omar Costilla-Reyes

**Affiliations:** a Massachusetts Institute of Technology, 77 Massachusetts Avenue, Cambridge, MA, 02139, USA; b Harvard Medical School, 25 Shattuck St, Boston, MA, 02115, USA; c Boston Children’s Hospital, 300 Longwood Avenue, Boston, MA, 02115, USA; d Brigham and Women’s Hospital, 75 Francis Street, Boston, MA, 02115, USA

**Keywords:** Mental health, machine learning, mood disorders, major depressive disorder, anxiety disorders, comorbidity

## Abstract

Comorbid anxiety disorders are common among patients with major depressive disorder (MDD), and numerous studies have identified an association between comorbid anxiety and resistance to pharmacological depression treatment. However, the impact of anxiety on the effectiveness of non-pharmacological interventions for MDD is not as well understood. In this study, we applied machine learning techniques to predict treatment responses in a large-scale clinical trial (*n* = 493) of individuals with MDD, who were recruited online and randomly assigned to one of three smartphone-based interventions. Our analysis reveals that a baseline GAD-7 questionnaire score in the moderate to severe range (> 10) predicts reduced probability of recovery from MDD. Our findings suggest that depressed individuals with comorbid anxiety face lower odds of substantial improvement in the context of smartphone-based therapeutic interventions for depression. Our work highlights a methodology that can identify simple, clinically useful “rules of thumb” for treatment response prediction using interpretable machine learning models.^1^

## 1. Introduction

Major depressive disorder (MDD) affects roughly 322 million people, and is a leading cause of disability with large impacts on quality of life (Moreno-Agostino et al., 2021). Less than 20% of people with MDD receive minimally adequate treatment (Thornicroft et al., 2017). 50% of patients who receive MDD treatment experience minimal or no improvement, with a subset of these not responding to multiple treatment attempts (Gaynes et al., 2020). Digital and smartphone-delivered psychotherapy interventions are one promising avenue to increase access to evidence-based MDD treatments (Linardon et al., 2024). Knowledge of the factors that determine which patients respond well to these treatments could inform personalized treatment selection and identify opportunities to improve treatment response with additional or modified interventions. However, studies attempting to identify these predictors have yielded inconsistent results (Sextl-Plötz et al., 2024).

In this study, we explored predictors of clinical response to smartphone-delivered MDD treatments in a large, publicly available clinical trial dataset (Arean et al., 2016). In the Brighten MDD clinical trial, participants were recruited online and randomized to one of three digital treatments: Project EVO, a serious game designed to bolster cognitive skills related to MDD; iPST, an app based on problem-solving therapy for MDD; and Health Tips, an app that suggests strategies to improve mood, and serves as an active control. The clinical trial analysis found that participants assigned to Project EVO had significantly greater PHQ-9 score improvements than Health Tips at 4 weeks after enrollment, while improvements between the iPST and Health Tips groups were not significantly different (Arean et al., 2016). Although the original study compares the efficacy of the three interventions, the participant-level factors related to the likelihood of MDD improvement have not been explored to the best of our knowledge. We applied interpretable machine learning techniques, coupled with a forward feature selection approach, to identify variables measured at baseline that predict greater or lesser odds of a clinical response during the treatment period.

## 2. Methods

### 2.1. Models and variables

We attempted to predict binary MDD treatment responses using five machine learning algorithms: logistic regression, support vector machine, decision tree, random forest, and k-nearest-neighbors. Treatment response was defined as a PHQ-9 score at 4 weeks of both < 10 and ≥ 50% reduced relative to baseline (Kroenke et al., 2001). We considered the following variables in the dataset as “features” that the models could use for prediction (> 0% percentages of missing data shown in brackets after each variable name):

- Demographics:
  - Age
  - Gender (collected as binary male/female)
  - Race/ethnicity (binarized to non-Hispanic White/all others)
  - Working/employment (binary yes/no)
  - Marital status (binarized to married/partnered or not)
  - Education (binarized to education beyond high school or not)
  - Satisfaction with level of income (binarized to responses of “can’t make ends meet” vs. responses indicating higher satisfaction) [54% missing]
- Questionnaire scores:
  - Patient Health Questionnaire (PHQ-9) at baseline (Kroenke et al., 2001) [0%, 19%, 29%, 31%, and 33% missing at baseline and weeks 1–4 respectively]
  - Generalized Anxiety Disorder-7 (GAD-7) (Spitzer et al., 2006) [4% missing]
  - Sheehan Disability Scale (SDS) (Sheehan, 1983) [3% missing]
  - IMPACT mania and psychosis screening (used as two binary variables: mania history and psychosis history) (Unützer et al., 2002; Arean et al., 2016) [4% missin
  - AUDIT alcohol consumption questions (AUDIT-C) (Bush et al., 1998) [3% missing]
- Treatment group (binary feature for each)
  - Project EVO
  - iPST
  - Health Tips (active control)

### 2.2. Data preparation

Data were downloaded from the Brighten Study Public Researcher Portal on Synapse.org. While demographics and baseline PHQ-9 scores were collected upon enrollment, several questionnaire scores were collected in the days to weeks following enrollment, notably GAD-7, SDS, AUDIT-C, and mania and psychosis history. For these questionnaires, we used the earliest available response from each participant as the “baseline” measurement. We did not consider any such responses that were made more than 2 weeks after enrollment. We used only records from the Brighten Version 1 study (Arean et al., 2016) and excluded any participants with a baseline PHQ-9 score below 10, leaving 754 eligible participants. We excluded 35% of these participants, who had only demographic data and baseline PHQ-9 (which were collected together), without any questionnaire scores for GAD-7, SDS, IMPACT, AUDIT-C, and all post-intake PHQ-9: this resulted in a final sample size of 493. Before training each machine learning model, all non-binary features in the dataset were rescaled to have a mean of 0 and a standard deviation of 1. We used a random forest-based multiple imputation strategy with predictive mean matching to handle missing data (baseline variables and follow-up PHQ-9 at weeks 1–4), implemented with the miceRanger package in R (Wilson, 2020). We produced 100 imputed versions of the dataset.

### 2.3. Feature selection and model fitting

To minimize overfitting due to the high number of features in the dataset, we used a forward selection procedure to identify a minimal set of input features for each machine learning model. AUC estimates were first obtained for univariate models on each feature, and the feature that resulted in the highest AUC was selected. Then, all possible bivariate models including the first chosen feature were tested, and features were added one by one in this fashion until adding another feature did not significantly increase AUC. Each AUC estimate was obtained by 10,000-fold cross-validation. For each fold, we first randomly selected 1 of the 100 imputed versions of the dataset, and then randomly sampled 80% of the participants to form a training set, leaving the remaining 20% as a validation set. We took the 10,000 difference values between estimates from the current candidate model and those of the previous best model (or AUC = 0.5 for the first variable). We computed both the mean ΔAUC value and its 95% confidence interval from this empirical distribution, which accounts for uncertainty due to both missingness and recruitment sampling. Statistical significance of the model’s improvement due to the added variable was assessed by calculating the probability *p* = *P*(ΔAUC ≤ 0) from the bootstrapped distribution. The Benjamini-Hochberg procedure (Benjamini and Hochberg, 1995) was applied to control the false discovery rate at 0.05 across all comparisons. To balance the goals of maximizing prediction accuracy and limiting model complexity, we also require an AUC improvement of ≥ 0.02 for each new variable (Greenberg et al., 2024). For decision trees and random forests, we run variable selection for different maximum tree depths (maximum number of binary decisions allowed per tree, ranging here from 1 to 5 inclusive), keeping the tree depth that produced the highest AUC estimate overall. All models were implemented using the SciKit-Learn Python library with default hyperparameters (Pedregosa et al., 2011).

## 3. Results

Random forests, decision trees, and logistic regression demonstrated AUC values significantly above the 0.5 chance level in predicting treatment response, while support vector machines and k-nearest-neighbors did not. Random forests reached marginally better performance (AUC = 0.652) than decision trees (AUC = 0.645) and logistic regression (AUC = 0.644). All selected models used only one feature, GAD-7: in no configuration did adding any additional variable increase AUC with statistical significance. Random forest and logistic regression models also predicted significantly above chance using SDS as the sole predictor (indicating a negative relationship between functional disability and depression response), but were not selected by the forward process due to marginally lower AUC estimates.

**Figure 1:**
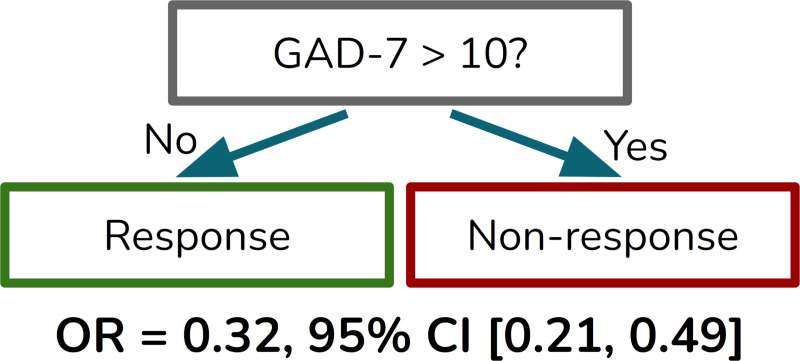
A decision tree fitted to the multiply-imputed dataset shows the importance of the baseline GAD-7 score in predicting MDD response. Participants who reported a GAD-7 score above 10 were roughly one-third as likely to experience significant MDD improvement as those with a score of 10 or below.

Decision trees have the advantage of simplicity (in the simplest case, a single binary decision on one variable), and perform comparably to logistic regression and random forests. Re-fitting a depth=1 decision tree on the entire dataset yields a decision tree that classifies participants with a GAD-7 score above 10 (in the “moderate” to “severe” range for generalized anxiety disorder (Spitzer et al., 2006)) as likely non-responders, and those with a GAD-7 score of 10 or below as likely responders (Fig. 1). To better quantify the association of GAD-7 being above 10 with MDD outcomes, we calculated an odds ratio of 0.32 for response vs non-response given GAD-7 > 10, with a 95% confidence interval of [0.21,0.49]. These findings indicate that a GAD-7 score above 10 reduces the odds of a clinical response by a factor of roughly one-third, with statistical significance. We used Rubin’s rules to combine the odds ratio values from multiple imputations (Rubin, 1987).

## 4. Discussion

We predicted treatment responses in a large cohort of participants who received smartphone-delivered treatments for MDD. Our decision tree analysis identified a clear and clinically meaningful relationship: depressed individuals with baseline GAD-7 scores above 10 (“moderate” to “severe” anxiety) were only about one third as likely to recover as those with lower GAD-7 scores.

Our findings highlight the utility of decision trees as a readily interpretable non-linear modeling approach. While decision trees demonstrated similar AUC scores to logistic regression and random forest models, these three model types offer different levels of interpretability - the ability to understand and explain the way the trained model makes predictions. Like many modern machine learning approaches (notably including deep neural networks), random forests are effectively uninterpretable ”black-boxes”: we cannot explain how our random forests use GAD-7 to predict outcomes. Our logistic regression models indicated that higher GAD-7 scores are associated with lower response rates, but the strength of this relationship must be quantified to allow clinicians to appropriately weigh its potential clinical impact, and logistic regression coefficients are less than intuitive. In contrast, decision trees provided a GAD-7 *threshold* (GAD-7 > 10) above which treatment response is less likely. While decision trees are commonly overlooked in both modern machine learning and traditional statistical analyses, they can generate predictive rules that can be easily interpreted by clinicians and thus directly inform clinical decision-making (Banerjee et al., 2019).

It is important to note two limitations of this study. As would be expected from a remotely-recruited national sample for an effectiveness trial, there is a substantial proportion of missing data. We address this limitation by using a rigorous multiple imputation approach. Secondly, our analysis cannot establish causal associations. One possible explanation for the association between baseline GAD-7 and MDD treatment response is that anxiety hinders participants’ engagement with smartphone-delivered treatments. Given findings from previous studies that comorbid anxiety reduces pharmacological treatment response in MDD (e.g., Fava et al. (2008); Saveanu et al. (2015); Dold et al. (2017)), it is also conceivable that comorbid anxiety hinders treatment response through a mechanism that is independent of treatment modality. A third possibility is that anxiety simply reduces the odds of recovery from MDD regardless of whether an individual is receiving treatment. Although the original clinical trial analysis found higher MDD remission rates for participants in the Project EVO and iPST groups compared to the Health Tips control group (Arean et al., 2016), our analysis did not identify treatment assignment as a significant predictor of outcome. Notably, however, we used different inclusion criteria and our analysis was designed to assess the strength of multiple predictor variables (with rigorous corrections for multiple comparisons), not treatment efficacy. Ultimately, our study cannot distinguish between treatment-related, treatment-unrelated, or combined mechanisms for the observed association between baseline GAD-7 scores and PHQ-9 score reductions.

Understanding the relationship between anxiety and MDD recovery may be clinically actionable depending on the mechanisms involved. For example, if patients with comorbid anxiety struggle to engage with smartphone-delivered treatments, they might require additional support or alternative treatment modalities. Regardless of the underlying mechanisms, one practical implication of our results is that it may be necessary to stratify group assignment by baseline anxiety levels in future randomized controlled trials for digital MDD interventions.

## Figures and Tables

**Table 1:** GAD-7 was the only predictor of treatment response across all selected models, as adding any second variable did not significantly increase AUC. Alternative models that predicted significantly above chance, but were not selected by the forward process, are shown in gray. Support vector machines and k-nearest-neighbors failed to predict significantly above chance (N.S. = not significant). Logistic regression coefficients are for standardized features (mean=0, std=1). Tree depth is only applicable for random forests and decision trees, and model coefficients are only applicable for logistic regression and support vector machines.

Model type	Interpretable?	AUC (95% CI)	Tree depth	Predictor(s)	Coef. (95% CI)
Random Forest	No	0.652 (0.55, 0.75)	2	GAD-7	-
		0.645 (0.54, 0.74)	1	GAD-7	-
		0.638 (0.53, 0.74)	1	SDS	-
		0.634 (0.53, 0.74)	2	SDS	-

Decision Tree	Yes	0.645 (0.55, 0.74)	2	GAD-7	-
		0.639 (0.54, 0.74)	3	GAD-7	-
		0.631 (0.54, 0.72)	1	GAD-7	-

Logistic Regression	Yes	0.644 (0.54, 0.74)	-	GAD-7	−0.52 (−0.65, −0.40)
		0.641 (0.54, 0.74)	-	SDS	−0.49 (−0.62, −0.37)

Support Vector Machine	Yes	N.S.	-	N.S.	N.S.

K-Nearest-Neighbors	No	N.S.	-	N.S.	-
