# Supplementary material for: Comorbid anxiety predicts lower odds of MDD improvement in a trial of smartphone-delivered interventions
Source: ArXiv. 2025 Aug 1:arXiv:2409.11183v3. Preprint. [Version 3] (PMC11838690)
Supplement: Supplement 1 [file NIHPP2409.11183v3-supplement-1.pdf]

Supplementary Material: Comorbid anxiety predicts lower odds of MDD improvement in a trial of smartphone-delivered interventions

Morgan B. Talbot[a,b,c,*], Jessica M. Lipschitz[b,d], Omar Costilla-Reyes[a]

[a]*Massachusetts Institute of Technology, 77 Massachusetts Avenue, Cambridge, MA, 02139, USA*
[b]*Harvard Medical School, 25 Shattuck St, Boston, MA, 02115, USA*
[c]*Boston Children's Hospital, 300 Longwood Avenue, Boston, MA, 02115, USA*
[d]*Brigham and Women's Hospital, 75 Francis Street, Boston, MA, 02115, USA*

1. Full table of selected and alternate models from main analysis

This section provides the complete results from our main analysis, supplementing the summary table in the main manuscript (Main Text Table 1). We tested five categories of classical machine learning algorithms: logistic regression, support vector machines, decision trees, random forests, and k-nearest-neighbors. For the tree-based models, we ran the analysis for maximum tree depths ranging from 1 to 5.

Supplementary Table 1 presents the performance metrics for all models that performed significantly above the chance level. The additional results shown here are fully consistent with the conclusions in the main text. For both decision trees and random forests, increasing the tree depth beyond 1 or 2 offered no substantive improvement in performance. Although deeper trees yielded marginally increased AUC point estimates, accuracy remained nearly identical across all depths. This occurs because AUC rewards models that assign more fine-grained probability scores, which deeper trees can achieve by partitioning the data into more groups. However, the stability of the accuracy metric suggests that a single decision threshold (as found in a depth=1 tree) is sufficient to capture the main predictive relationship in this dataset.

2. Sensitivity analyses

We conducted alternate versions of our main analysis to assess the sensitivity of our findings to certain assumptions and data pre-processing decisions. Specifically, we tested sensitivity to the timing of the Patient Health Questionnaire-9 (PHQ-9) used to measure outcomes, and to participant exclusion criteria based on data missingness.

2.1. Sensitivity to timing of endpoint PHQ-9 measurement

In the main analysis for our study, we used a PHQ-9 measurement 4 weeks after trial enrollment to assess MDD outcomes. Participants were considered to have experienced MDD improvement if their PHQ-9 score on

*Corresponding author at: Department of Health Sciences and Technology, Massachusetts Institute of Technology, 77 Massachusetts Avenue Building E25-518, Cambridge, MA 02139, USA.

Email address: `mtalbot@mit.edu` (Morgan B. Talbot)

Model Type	Interp?	AUC	Accuracy	Depth	Pred.	Coefficient
Logistic Regression	Yes	0.74 (0.65, 0.82)	0.69 (0.61, 0.77)	-	GAD-7	-0.96 (-1.13, -0.80)
		0.71 (0.62, 0.79)	0.66 (0.58, 0.74)	-	SDS	-0.75 (-0.93, -0.57)
Support Vector Machine	Yes	0.74 (0.65, 0.82)	0.70 (0.62, 0.78)	-	GAD-7	-1.01 (-1.15, -0.88)
		0.70 (0.61, 0.79)	0.66 (0.57, 0.74)	-	SDS	-0.81 (-0.98, -0.58)
Random Forest	No	0.74 (0.65, 0.82)	0.70 (0.62, 0.78)	2	GAD-7	-
		0.73 (0.64, 0.81)	0.70 (0.62, 0.78)	3	GAD-7	-
		0.73 (0.65, 0.81)	0.70 (0.62, 0.78)	1	GAD-7	-
		0.73 (0.64, 0.81)	0.70 (0.62, 0.77)	4	GAD-7	-
		0.73 (0.64, 0.81)	0.69 (0.61, 0.77)	5	GAD-7	-
		0.70 (0.61, 0.79)	0.66 (0.58, 0.74)	2	SDS	-
		0.70 (0.61, 0.78)	0.66 (0.57, 0.74)	1	SDS	-
		0.69 (0.60, 0.78)	0.66 (0.57, 0.73)	3	SDS	-
		0.69 (0.60, 0.78)	0.65 (0.56, 0.73)	4	SDS	-
		0.69 (0.59, 0.77)	0.65 (0.56, 0.73)	5	SDS	-
Decision Tree	Yes	0.73 (0.64, 0.81)	0.70 (0.61, 0.77)	3	GAD-7	-
		0.73 (0.64, 0.81)	0.69 (0.61, 0.77)	4	GAD-7	-
		0.72 (0.64, 0.80)	0.70 (0.62, 0.78)	2	GAD-7	-
		0.72 (0.64, 0.81)	0.69 (0.61, 0.77)	5	GAD-7	-
		0.70 (0.61, 0.77)	0.69 (0.61, 0.77)	1	GAD-7	-
		0.69 (0.60, 0.77)	0.65 (0.57, 0.73)	3	SDS	-
		0.68 (0.60, 0.77)	0.65 (0.57, 0.73)	2	SDS	-
		0.68 (0.59, 0.77)	0.65 (0.56, 0.73)	4	SDS	-
		0.68 (0.59, 0.77)	0.65 (0.56, 0.73)	5	SDS	-
		0.65 (0.56, 0.73)	0.64 (0.55, 0.73)	1	SDS	-
K-Nearest-Neighbors	No	N.S.	N.S.	-	N.S.	-

Table 1: **Performance results for all models predicting significantly above chance during forward variable selection in the main analysis.** The format of this table matches that of Main Text Table 1, which contains a subset of the results shown here. Values in parentheses are 95% confidence intervals. "Interp." indicates whether each model type is considered interpretable. Coefficients for logistic regression and support vector machine models are for standardized features with mean=0 and std=1. "Depth" indicates the maximum tree depth for tree-based models. Alternative models that predicted significantly above chance, but were not selected by the forward process, are shown in gray.

post-enrollment week 4 was both less than 10 and reduced by at least 50% relative to baseline. In this sensitivity analysis, we apply the same MDD improvement criterion to PHQ-9 measurements on post-enrollment week 12. In the original Brighten trial, PHQ-9 scores were measured at baseline and on weeks 1, 2, 3, 4, 6, 8, 10, and 12 (Arean et al., 2016). The missingness of PHQ-9 measurements on these weeks respectively is as follows: 52%, 58%, 59%, 60%, 67%, 72%, 75%, 77%. Like in our main analysis, we employ a random-forest based multiple imputation approach to impute missing values. In this case, we include PHQ-9 values at all weeks (i.e., up to week 12) in the imputation procedure.

Supplementary Table 2 shows the full results of this sensitivity analysis, which are highly similar to those of our main analysis. Generalized Anxiety Disorder-7 (GAD-7) remains the most important predictor, with SDS yielding alternative, statistically significant but less performant models of all types. Both AUC and accuracy for models using GAD-7 fall within narrow ranges across all model types, with depth=1 decision trees performing equivalently to trees with higher maximum depth values. Like in the main analysis, we fitted depth=1 decision trees on every imputed version of the dataset and took the median GAD-7 threshold. This yielded the same prediction rule of GAD-7 $\geq$ 11 predicting lower odds of MDD improvement, with an odds ratio of 0.27 (95% CI [0.17, 0.43], $p < 0.0001$). The results of this sensitivity analysis indicate that GAD-7 $\geq$ 11 predicts lower odds of MDD improvement in this trial on a 12-week time scale in addition to a 4-week time scale.

2.2. Sensitivity to exclusion of participants with minimal data

Of the participants included in our main analysis, 41% were missing all baseline and post-baseline clinical questionnaire data aside from baseline PHQ-9. While these participants were assigned to receive one of three smartphone-delivered interventions in the original Brighten study (Anguera et al., 2016; Arean et al., 2016), the only available data from them are demographic variables and baseline PHQ-9. These participants are the primary source of missingness in the overall dataset. To evaluate the effect of relatively extensive imputation of missing values, we conducted a sensitivity analysis that excluded this subset of participants, with the caveat that this approach has the potential to introduce selection biases. Participants were included in this analysis if they met the following criteria:

1. Baseline PHQ-9 of 10 or higher (same as main analysis)
2. No endorsed history consistent with bipolar disorder (same as main analysis)
3. At least one of the following measurements: AUDIT alcohol consumption questions (AUDIT-C, at baseline), GAD-7 (at baseline), IMPACT Mania and Psychosis Screening (at baseline), Sheehan Disability Scale (SDS, at baseline), or PHQ-9 at 1, 2, 3, or 4 weeks post-enrollment.

The third criterion above excluded 279 (41%) of the 638 otherwise eligible participants who were included in our main analysis, leaving a sample size of $n = 359$. This sensitivity analysis used the same set of variables as the main analysis for imputation, variable selection, and outcome prediction. The missingness of the dataset used for this sensitivity analysis is as follows: 54% missing satisfaction with level of income, 10% missing AUDIT-C, 11% missing GAD-7,

Model Type	Interp?	AUC	Accuracy	Depth	Pred.	Coefficient
Logistic Regression	Yes	0.68 (0.59, 0.77)	0.64 (0.55, 0.72)	-	GAD-7	-0.65 (-0.85, -0.50)
		0.66 (0.56, 0.75)	0.61 (0.52, 0.70)	-	SDS	-0.58 (-0.76, -0.41)
Support Vector Machine	Yes	0.68 (0.59, 0.77)	0.63 (0.55, 0.71)	-	GAD-7	-0.84 (-1.00, -0.72)
		0.66 (0.56, 0.75)	0.61 (0.52, 0.69)	-	SDS	-0.81 (-0.92, -0.70)
Random Forest	No	0.67 (0.58, 0.76)	0.65 (0.56, 0.73)	2	GAD-7	-
		0.67 (0.58, 0.76)	0.65 (0.56, 0.73)	1	GAD-7	-
		0.67 (0.57, 0.76)	0.64 (0.56, 0.73)	3	GAD-7	-
		0.66 (0.57, 0.76)	0.64 (0.55, 0.72)	4	GAD-7	-
		0.66 (0.57, 0.76)	0.63 (0.55, 0.72)	5	GAD-7	-
		0.65 (0.56, 0.74)	0.60 (0.52, 0.69)	2	SDS	-
		0.65 (0.56, 0.74)	0.60 (0.52, 0.69)	1	SDS	-
		0.64 (0.54, 0.73)	0.60 (0.51, 0.68)	3	SDS	-
		0.63 (0.54, 0.72)	0.59 (0.50, 0.68)	4	SDS	-
Decision Tree	Yes	0.66 (0.57, 0.75)	0.64 (0.55, 0.73)	3	GAD-7	-
		0.66 (0.57, 0.75)	0.63 (0.55, 0.72)	5	GAD-7	-
		0.66 (0.57, 0.74)	0.64 (0.56, 0.73)	2	GAD-7	-
		0.66 (0.57, 0.75)	0.63 (0.55, 0.72)	4	GAD-7	-
		0.65 (0.56, 0.73)	0.65 (0.56, 0.73)	1	GAD-7	-
		0.63 (0.54, 0.72)	0.59 (0.51, 0.68)	3	SDS	-
		0.63 (0.54, 0.72)	0.60 (0.51, 0.68)	2	SDS	-
K-Nearest-Neighbors	No	N.S.	N.S.	-	N.S.	-

Table 2: **Performance results for all models predicting significantly above the chance level in a sensitivity analysis, in which MDD outcomes were predicted at week 12 instead of week 4.** The format of this table is identical to Main Text Table 1 and Supplementary Table 1.

11% missing SDS, and 0%, 20%, 30%, 31%, and 33% missing PHQ-9 at baseline and weeks 1-4 respectively. All other variables had 0% missingness.

The model performance results of this sensitivity analysis are shown in Supplementary Table 3. Only one model predicted significantly above chance, a depth=2 random forest using GAD-7 as the sole predictor. It is likely that other models failed to cross the significance threshold because of the considerably reduced sample size relative to the main analysis. Although it did not survive the correction for multiple comparisons with statistical significance, we examine the depth=1 decision tree using GAD-7 as a predictor due to its central role in our main findings. This model had a mean cross-validated AUC of 0.63 (95% CI: [0.53, 0.74], $p = 0.007$, not significant), with mean accuracy 0.63 (95% CI: [0.51, 0.74]). As in the main analysis, we fitted depth=1 decision trees to each imputed dataset version, finding the median GAD-7 threshold to yield the same GAD-7 ≥ 11 decision rule as our main results. In this sensitivity analysis, GAD-7 ≥ 11 predicts lower odds of MDD improvement with odds ratio 0.30 (95% CI [0.18, 0.49], $p < 0.0001$).

While the results of this sensitivity analysis did not find evidence for a depth=1 decision tree at the level of variable selection, a random forest model using GAD-7 predicted significantly above chance even under correction for multiple comparisons. Furthermore, testing the GAD-7 hypothesis generated by forward selection in our main analysis produced the same GAD-7 ≥ 11 threshold and yielded a highly significant odds ratio. While this should be interpreted with caution as an exploratory finding, the overall results of this sensitivity analysis are consistent with those of the main analysis.

**Model Type**	**Interp?**	**AUC**	**Accuracy**	**Depth**	**Pred.**	**Coefficient**
Logistic Regression	Yes	N.S.	N.S.	-	N.S.	N.S.
Support Vector Machine	Yes	N.S.	N.S.	-	N.S.	N.S.
Random Forest	No	0.67 (0.55, 0.78)	0.64 (0.54, 0.75)	2	GAD-7	-
Decision Tree*	Yes	0.63 (0.53, 0.74)*	0.63 (0.51, 0.74)	1	GAD-7	-
K-Nearest-Neighbors	No	N.S.	N.S.	-	N.S.	-

Table 3: **Performance results for all models predicting significantly above chance in a sensitivity analysis, which excluded participants with only baseline demographic data and no questionnaire data beyond baseline PHQ-9.** The format of this table is identical to Main Text Table 1 and Supplementary Table 1. *The model marked with an asterisk did not meet the statistical significance threshold for forward selection in this sensitivity analysis (under correction for multiple comparisons), but is shown for its relevance to the hypothesis generated in the main analysis.

References

Anguera, J.A., Jordan, J.T., Castaneda, D., Gazzaley, A., Areán, P.A., 2016. Conducting a fully mobile and randomised clinical trial for depression: access, engagement and expense. BMJ innovations 2.

Arean, P.A., Hallgren, K.A., Jordan, J.T., Gazzaley, A., Atkins, D.C., Heagerty, P.J., Anguera, J.A., 2016. The use and effectiveness of mobile apps for depression: results from a fully remote clinical trial. Journal of Medical Internet Research 18, e330.
